# Challenging the guidelines: longitudinal trends in left ventricular diameter and function in severe aortic regurgitation

**DOI:** 10.3389/fcvm.2026.1975473

**Published:** 2026-10-22

**Authors:** Shmuel Schwartzenberg, Amit Berkowitz, Tsahi T. Lerman, Tamir Bental, Mordehay Vaturi, Yair Goldberg, Yaron Shapira

**Affiliations:** 1 The Department of Cardiology, Rabin Medical Center, Beilinson Hospital, Petah Tikva, Israel; 2 Affiliated with the Gray Faculty of Medical & Health Sciences, Tel-Aviv University, Tel Aviv, Israel; 3 The Faculty of Data and Decision Sciences, Technion – Israel Institute of Technology, Haifa, Israel

**Keywords:** aortic regurgitation, fractional shortening, joint model analysis, left ventricular end diastolic diameter, left ventricular end systolic diameter

## Abstract

**Background:**

Guidelines recommend aortic valve replacement (AVR) in patients with severe aortic regurgitation (AR) based on progressive changes in left ventricular (LV) function or size. We aimed to reassess the clinical relevance of current guideline recommendations pertaining to traditional echocardiographic measurements in routine practice.

**Methods:**

Retrospective analysis of patients with severe AR who underwent serial echocardiographic follow-up over at least 18 months. The composite outcome was symptom-driven AVR, acute heart failure hospitalization, or death. We used a joint modelling approach to handle within-subject correlation and censoring.

**Results:**

The cohort consisted of 140 patients, with a median follow-up of 93 months (interquartile range 58–130). LV end-systolic (LVESD) and fractional shortening (FS) showed a small but statistically significant longitudinal trend, while LVEDD did not. In three separate joint models adjusted for age at onset and sex, higher LVESD and LVEDD and lower FS were each associated with increased risk of the composite event. Each 1 mm increase in LVESD and LVEDD was associated with a 6% in risk; each 1% decrease in FS corresponded to a 12% increase in risk. Only 8 (5.7%) of patients were predicted to exceed the guideline-recommended LVEDD threshold of 65 mm over 10 years. Age at onset was also a significant risk factor, with each decade increasing risk by 65% for each of the three parallel joint models.

**Conclusion:**

LV parameters show modest changes over time, despite holding strong prognostic value in patients with severe AR. LVEDD, while associated with overall risk, does not predictably or significantly dilate over time in most patients. AVR decisions should be based on comprehensive clinical and volumetric assessment rather than waiting for simple linear progression to guideline cutoffs.

## Graphical Abstract

**Figure:**
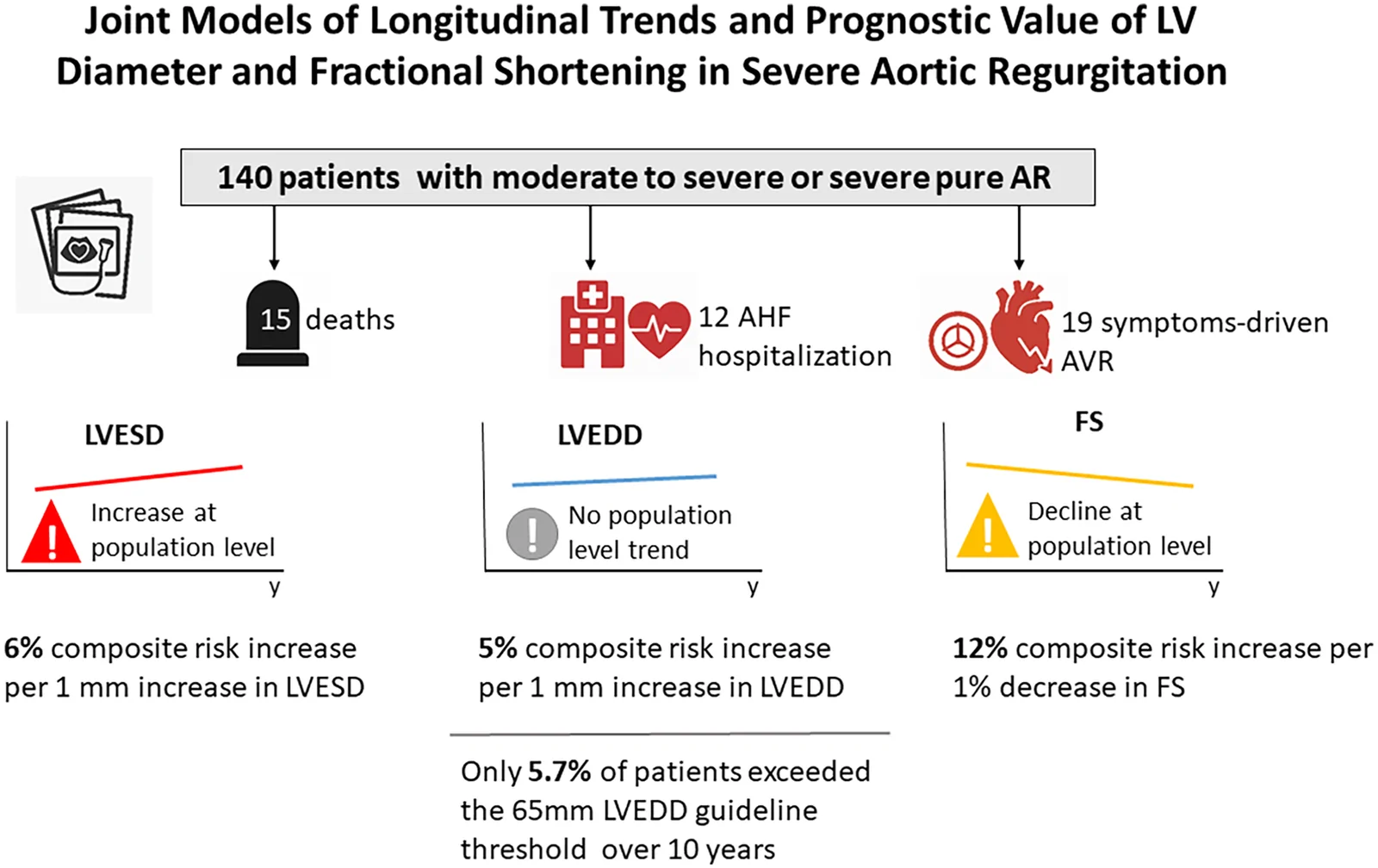

## 1 Introduction

Chronic severe aortic regurgitation (AR) leads to left ventricle (LV) volume overload, resulting in progressive eccentric hypertrophy and dilatation to counter high LV wall stress (1). Recommendations for aortic valve replacement (AVR) related to the severity of LV dilation in AR are based on transthoracic echocardiography (TTE) parasternal long-axis linear measurement of LV at base and LV ejection fraction (LVEF) (2, 3). A LVEF less than 55% (per ACC/AHA) (2) or less than 50% (per ESC/EACTS) (3) carries a strong (Class I) indication for AVR. An LV end systolic diameter (LVESD) greater than 50 mm, or an indexed LVESD greater than 25 mm/m^2^, is also considered a Class I indication for AVR per ESC/EACTS guidelines, or Class IIa per ACC/AHA guidelines, and is well supported by previous studies (4–8).

Current valve guidelines suggest that AVR may be considered in patients with severe AR based on progressive changes in left ventricular function or size, including: (1) a progressive decline in LVEF on at least three serial studies, reaching the low–normal range (55%–60%) (2); and (2) a progressive increase in LV end-diastolic diameter (LVEDD) above 65 mm (2, 3). However, to our knowledge, only two old studies by a single research group have actually looked into how these parameters change over time in patients with severe AR, and whether such changes are associated with adverse outcomes. A 1980 study by Henry et al. followed 37 patients with repeated echocardiographic exams over a mean of 34 months (9). A later study, published in 1991 by Bonow et al., analyzed serial echocardiographic and radionuclide angiographic studies over a mean of 8 years in 104 patients with severe AR and normal LVEF (8). In the 1991 study, the rate of LVEDD progression did not differ between patients who remained stable and those who experienced adverse outcomes—specifically, death, heart failure symptoms, or deterioration in LV systolic function (0.3 ± 0.8 vs. 0.4 ± 0.8 mm/year, respectively). However, LVESD increased more in the event group (1.1 ± 1.3 vs. 0.2 ± 0.7 mm/year, *p* < 0.005) (8). In the earlier study, the average LVESD change was 2.6 ± 1.2 mm/year (9).

Given the gaps outlined above, we aimed to reassess the clinical relevance of current guideline recommendations pertaining to changes in standard echocardiographic measures. Specifically, we examined how LVEDD, LVESD, and fractional shortening (FS) change over time in patients with pure chronic moderate to severe AR or greater. Moreover, we assessed whether these measures are associated with adverse clinical outcomes.

## 2 Methods

### 2.1 Study population

We identified all consecutive patients with moderate-to-severe or severe AR on baseline transthoracic echocardiography (TTE) and at least three follow-up TTEs over more than 18 months at a large tertiary medical center between April 1994 and April 2024. Exclusion criteria were concomitant greater than mild to moderate aortic stenosis or mitral valve disease, aortic dissection, prior valve surgery, hypertrophic cardiomyopathy, complex congenital heart disease, or LV assist devices (Figure 1). The primary composite outcome included symptom-driven AVR, acute heart failure hospitalization, or all-cause mortality.

**Figure 1:**
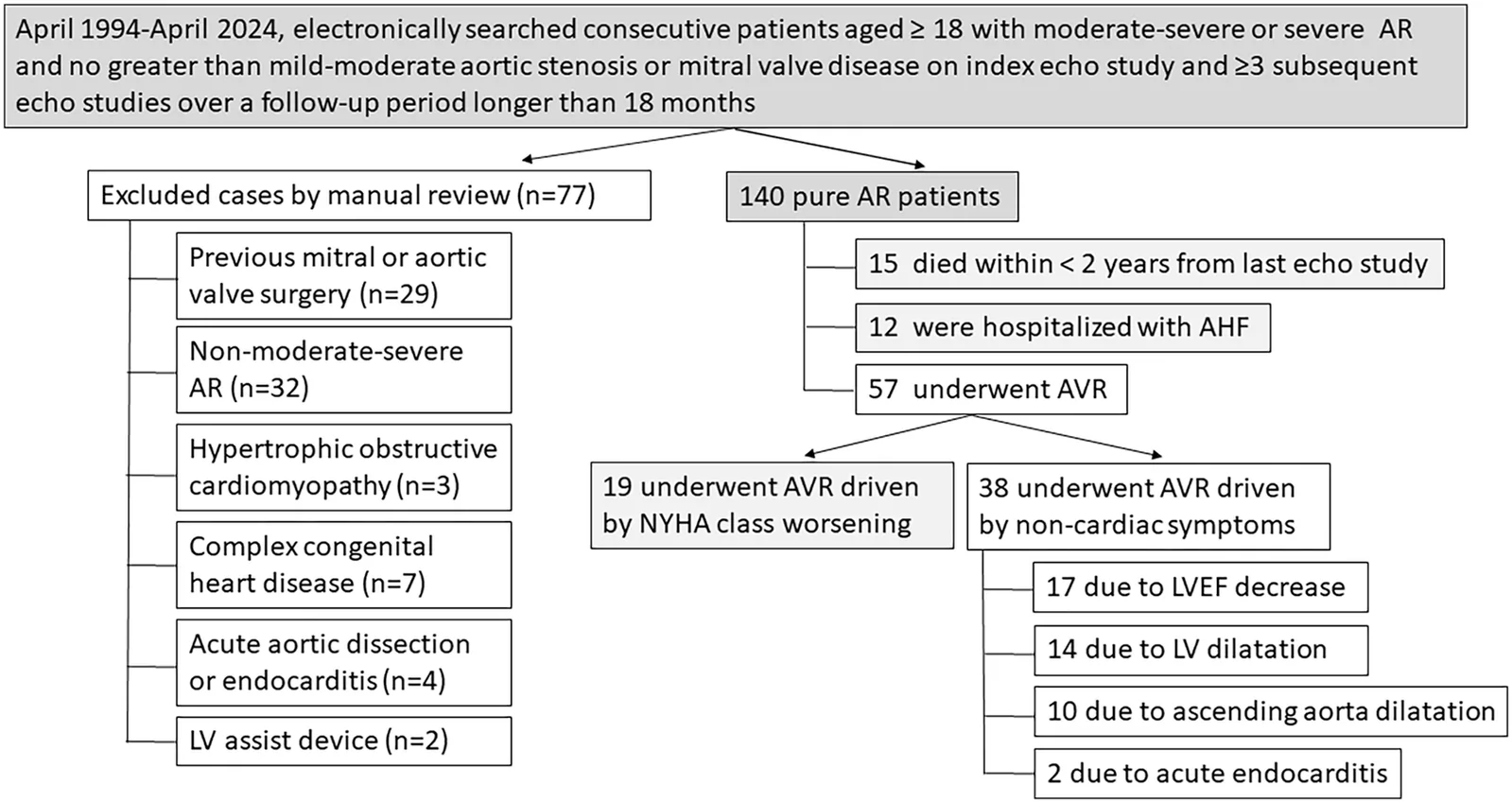
Study flow.

We extracted baseline characteristics and echocardiographic measurements for all patients from electronic medical records and echo reports, and the causes for AVR from the medical visit records. The study was approved by the hospital's Institutional Review Board.

### 2.2 Echocardiography studies

All patients underwent comprehensive echocardiograms using commercial instruments. All TTE studies were performed according to the guidelines of the American (2, 10, 11) and European cardiology societies (3, 12). Morphology of AV (trileaflet vs. other) was documented. The inner LV cavity diameter was measured at end-diastole and end-systole at the traditional basal ventricular level on parasternal long axis view. Fractional shortening (FS) was calculated by the formula (LVEDD-LVESD)*100/LVEDD.

### 2.3 Clinical endpoints

To capture major clinically consequential outcomes during longitudinal follow-up, the primary composite endpoint was defined as the time to the first occurrence of all-cause death, acute heart failure hospitalization, or worsening heart failure (HF) symptoms prompting AVR.

Worsening symptoms were defined as a deterioration of at least one New York Heart Association (NYHA) functional class despite medical management, prompting AVR, either surgical or transcatheter replacement. The definition of AHF hospitalization was standardized according to societal guidelines, following a comprehensive examination and review of electronic medical records for clinical events.

Patients who did not experience any of these events were censored at the end of follow-up or at the time of AVR if it was performed for reasons other than the appearance of cardiac symptoms. Such reasons included a decrease in LVEF, an increase in LVESD or indexed LVESD, MRI-determined LV enlargement, or dilatation of the ascending aorta or aortic root beyond accepted thresholds at the time of their referral to surgical treatment.

### 2.4 Statistical analysis

We applied an analysis that combines the following two key aspects: (1) characterizing how each patient's echocardiographic measures, specifically FS, LVESD, and LVEDD change over time; and (2) assessing how their underlying values are related to the risk of experiencing a composite event during follow-up. By fitting these two aspects together, the analysis can reveal whether specific patterns in these measures provide an early indication of future risk. We used a *joint modeling approach* implemented in the JMbayes2 package (version 0.5.7) in R (13–15), linking a linear mixed-effects model for the repeated echocardiographic measurements with a Cox proportional hazards model for time-to-event data. Separate joint models were constructed for FS, LVESD, and LVEDD, adjusting for sex and age at onset in both submodels. Age at onset was defined as the patient's age at the first qualifying (baseline) TTE and was centered at the cohort median of 56 years.

Separate models were fitted because FS is calculated from LVEDD and LVESD and therefore is not independent of these measures. Simultaneously modeling all three markers would introduce this structural dependency and require estimation of a multivariate longitudinal process, including the correlations among marker-specific trajectories, together with multiple marker–outcome association parameters. Given the cohort size (140 patients) and 46 composite events, such a model would be insufficiently parsimonious for stable estimation. We therefore fitted separate joint models to estimate marker-specific associations with the composite outcome; these associations should not be interpreted as mutually adjusted or independent effects.

Each longitudinal submodel included follow-up time in months, sex, and centered age at onset as fixed effects, together with patient-specific random intercepts and slopes for time. Sex and age at onset were included in the longitudinal submodel to account for systematic differences in marker levels between patients. These random effects allowed both the initial marker level and its rate of change to vary between patients and were assumed to follow a bivariate normal distribution with an unstructured covariance matrix. Measurement errors were assumed to be normally distributed with constant variance and independent conditional on the random effects. The longitudinal analyses included 1,440 FS measurements, 1,440 LVESD measurements, and 1,471 LVEDD measurements.

The survival submodel was linked to the longitudinal submodel through the current underlying marker value. Specifically, the log hazard depended linearly on the patient-specific marker value at the same time, estimated by the longitudinal submodel while accounting for measurement error. Sex and age at onset were included in the survival submodel to account for their direct associations with the hazard of the composite outcome, independently of the current underlying marker value. Accordingly, the hazard ratios describe the association between the current underlying marker value and the hazard of the first occurrence of any component of the composite endpoint, conditional on gender and age at onset. The log baseline hazard was modeled using penalized quadratic B-splines with equally spaced knots, the package-default setting of 10 baseline-hazard segments, and a second-order difference penalty.

Bayesian estimation used the package-default prior distributions: normal priors for regression and association coefficients, gamma priors for random-effect and residual standard deviations, an LKJ prior for the random-effects correlation matrix, and a normal smoothing prior for baseline-hazard spline coefficients with a gamma prior on the smoothing precision. MCMC sampling was conducted using three chains of 15,000 iterations each, with the first 5,000 iterations discarded as burn-in and every fifth remaining iteration retained, yielding 6,000 posterior draws in total. Convergence was assessed using Gelman–Rubin diagnostics, which indicated good convergence for the parameters of primary interest.

Uncertainty in joint-model estimates was summarized using 95% equal-tailed credible intervals (CrIs), defined by the 2.5th and 97.5th percentiles of the posterior distribution. Reported *P* values were two-sided posterior tail probabilities calculated by JMbayes2 as twice the smaller proportion of retained posterior draws below zero or at or above zero for the corresponding coefficient. For hazard ratios, the corresponding reference value is one.

As an event-specific descriptive analysis, we summarized the first and last available echocardiographic measurements before the first event and the within-patient difference between them, separately according to the type of first event.

## 3 Results

### 3.1 Cohort characteristics

We included a cohort of 140 patients meeting the inclusion criteria on initial echo study after exclusion of 77 patients (Figure 1). The cohort had a median follow-up of 93 months (IQR 58–130) and a median of 9 TTE studies (IQR 6–13.5).

Table 1 presents baseline characteristics for the overall cohort and descriptively by observed composite-event status at the end of available follow-up. Aortic valve morphology was tricuspid in 49%, bicuspid in 28%, and other/unknown in the remainder. The median of age at onset was 56 years (IQR 40–70) and 83% were male. During follow-up, 46 (33%) patients experienced the composite outcome.

**Table 1:** Baseline data.

Variable	Overall (*n* = 140)	Censored (*n* = 94)	Event (*n* = 46)
Follow up in months	93 [58, 132]	95 [62, 150]	92 [57, 126]
Number of echo studies	9.5 [6.0, 14.0]	10.0 [7.0, 15.0]	9.0 [6.0, 12.0]
Age at onset (years)	56 [40, 70]	52 [37, 66]	70 [51, 76]
Male sex	116 (83%)	79 (84%)	37 (80%)
Body surface area (m^2^)	1.91 ± 0.20	1.93 ± 0.21	1.87 ± 0.19
Echocardiographic measurements at baseline			
AV type			
Tricuspid	68 (48.6%)	41 (43.6%)	27 (58.7%)
Bicuspid	39 (27.8%)	34 (36.2%)	5 (10.9%)
Unicuspid	4 (2.9%)	2 (2.1%)	2 (4.3%)
Quadricuspid	1 (0.7%)	1 (1.1%)	0 (0%)
Unknown	28 (20%)	16 (17%)	12 (26.1%)
Ascending aorta diameter (cm)	3.97 ± 0.67	3.92 ± 0.69	4.13 ± 0.57
LVEDD (mm)	57 ± 7	56 ± 6	58 ± 8
LVESD (mm)	37 ± 7	37 ± 6	38 ± 9
Interventricular septum (cm)	1.12 ± 0.22	1.11 ± 0.20	1.15 ± 0.25
Posterior wall (cm)	1.04 ± 0.18	1.03 ± 0.18	1.05 ± 0.19
FS (%)	35 ± 7	35 ± 6	36 ± 9
LV ejection fraction (EF)			
Good EF (55%–65%)	116 (82.9%)	84 (89%)	32 (69.5%)
Preserved EF (50%–54%)	12 (8.6%)	4 (4.3%)	8 (17.4%)
Mildly reduced EF (45%–49%)	10 (7.1%)	6 (6.4%)	4 (8.7%)
Mild to moderate EF reduction (40%–45%)	1 (0.7%)	0 (0%)	1 (2.2%)
Unknown	1 (0.7%)	0 (0%)	1 (2.2%)
RV systolic pressure (mm Hg)	24 [21, 30]	25 [21, 30]	22 [20, 30]
Mitral regurgitation grade			
None or mild	122 (87.2%)	80 (85.1%)	42 (91%)
Mild to moderate	17 (12.1%)	13 (13.8%)	4 (8.7%)
Unknown	1 (0.7%)	1 (1.1%)	0 (0%)
Aortic stenosis grade			
None or mild	138 (99%)	93 (99%)	45 (98%)
Mild to moderate	2 (1.4%)	1 (1.1%)	1 (2.2%)
Tricuspid regurgitation grade			
None or mild	131 (93.6%)	87 (92.5%)	44 (95.6%)
Mild to moderate	7 (5%)	6 (6.4%)	1 (2.2%)
Unknown	2 (1.4%)	1 (1.1%)	1 (2.2%)

Approximately normal variables are expressed as means ± standard deviations. Non-Gaussian continuous variables are expressed as median [interquartile range]. Categorical variables are expressed as counts (percentages).

Forty-six patients (33%) experienced the composite event: 15 deaths, 19 symptom-driven AVRs, and 12 HF hospitalizations. Thirty-eight additional AVRs occurred for non-symptomatic indications (e.g., LV dilation, LVEF decline, aortic enlargement) and were therefore censored. The AVR causes in these censored patients are detailed in Figure 1.

To complement the primary joint-model analysis, which used all repeated measurements to estimate their association with the composite outcome, we performed a descriptive analysis stratified by the type of first event. Table 2 summarizes the first and last observed echocardiographic measurements and the within-patient changes between them for all patients experiencing the composite outcome and separately for each component. Median FS decreased before all three event types, while the increase in LVESD was most pronounced among patients whose first event was HF hospitalization. LVEDD showed no consistent direction of change across event types.

**Table 2:** Observed echocardiographic changes before the first composite-outcome event.

	Composite Event	AHF hospitalization	Symptom-driven AVR	Death
	*n* = 46	*n* = 12	*n* = 19	*n* = 15
FS, %				
First	36.0 [31.1, 41.4]	35.3 [30.6, 41.2]	39.6 [31.7, 42.4]	35.6 [32.5, 38.6]
Last	33.3 [25.1, 35.4]	27.1 [16.3, 35.2]	34.5 [28.1, 37.0]	33.3 [26.4, 34.8]
Change	−5.95 [−11.7, 0]	−9.0 [−15.5, −0.4]	−6.9 [−10.8, −0.2]	−4.8 [−7.3, 2.2]
LVESD, mm				
First	37.5 [34.0, 40.75]	38.0 [34.5, 40.5]	35.0 [32.0, 40.5]	39.0 [35.0, 40.5]
Last	39.5 [34.0, 44.8]	46.0 [38.2, 53.8]	39.0 [33.5, 41.5]	39.0 [33.5, 43.0]
Change	3 [−1, 8]	11.0 [0.0, 13.5]	3.0 [−1.0, 5.0]	1.0 [−4.5, 4.5]
LVEDD, mm				
First	59 [55.0, 61.8]	59.5 [55.8, 62.2]	58.0 [52.5, 61.0]	60.0 [58.0, 61.0]
Last	56.5 [52.0, 62.0]	61.0 [56.5, 65.2]	55.0 [51.0, 61.0]	57.0 [50.5, 63.5]
Change	−0.5 [−4, 4]	2.5 [−0.5, 7.0]	−2.0 [−4.0, 2.5]	0.0 [−10.0, 5.0]
Timing				
Last TTE to event, months		6.3 [2.5, 7.9]	5.4 [3.9, 11.6]	8.4 [3.5, 11.9]

### 3.2 Joint model using fractional shortening (FS) as the longitudinal variable

Table 3 summarizes the joint model results with FS as the main echocardiographic measure. The longitudinal model accounts for repeated measurements from each patient and estimates how FS is affected by time, age at onset, and sex. In this analysis, a linear trend is fitted to each individual's FS values over time, and Table 3.I shows the population-average effects across patients.

**Table 3:** Joint model for fractional shortening (FS).

Variable	Mean	StDev	2.5%	97.5%	*P*
	I. Longitudinal submodel results				
Female aged 56 years at onset (%)	36.40	0.99	34.39	38.32	<0.01
Follow-up time (months)	−0.02	0.00	−0.03	−0.01	<0.01
Age at onset, centered at 56 years	−0.03	0.02	−0.08	0.01	0.17
Male sex (vs. female)	−1.62	1.07	−3.74	0.48	0.13
Residual SD (sigma)	5.09	0.10	4.89	5.30	<0.01
	II. Survival submodel results				
Age at onset (years)	0.05	0.01	0.02	0.07	<0.01
Male sex (vs. female)	−0.51	0.42	−1.28	0.37	0.23
Current FS (%)	−0.12	0.04	−0.19	−0.04	<0.01

For female patients aged 56 years, the predicted FS at baseline TTE was 36.40%. For male patients of the same age, the corresponding predicted FS was 34.78%, reflecting an estimated difference of −1.62 percentage points; however, this difference was not statistically significant. Over time, FS tended to decline significantly but slightly, by about 0.02% per month. Age at onset was not significantly associated with FS. The variability of individual FS measurements around their expected trends, represented by the sigma value, was approximately 5%. Overall, FS declined modestly over time across the population; however individual patterns varied widely, and no clear differences were observed by age at onset or gender.

Figures 2, 3 illustrate the observed and model-estimated FS trajectories, respectively, for four representative patients. The four patients were selected at random for illustration, with one patient drawn from each first-event category (death, symptom-driven AVR, and acute heart failure hospitalization) and one from the censored group. Figure 2 presents the observed FS measurements with an individual linear trend line shown for reference. In Figures 2A–C, the vertical dashed line indicates the timing of the clinical event; the patient shown in Figure 2D did not experience an event and was censored at the end of follow-up. Figure 3 presents the corresponding subject-specific FS trajectories estimated by the longitudinal submodel (blue), together with the fixed-effect reference trajectory for a female patient aged 56 years (black). The shaded illustrate residual measurement variability around each fitted subject-specific trajectory and were calculated as the fitted trajectory ±1.96 residual standard deviations.

**Figure 2:**
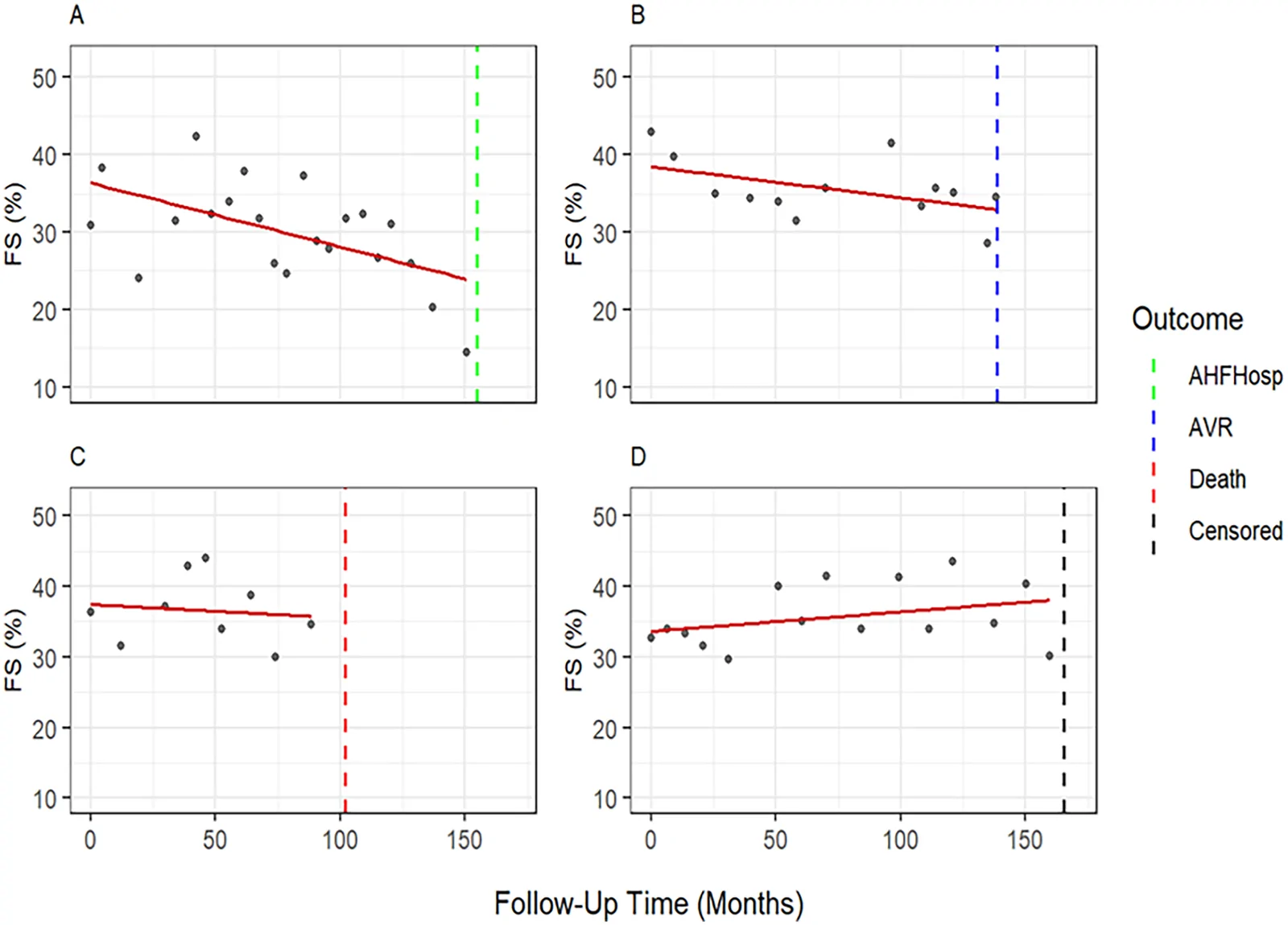
Observed fractional shortening trajectories in four illustrative patients. Observed FS measurements are shown as points; the red line in each panel is the patient-specific best-fitting linear trend, shown for descriptive purposes only. In panels **A–C**, the coloured vertical dashed line indicates the timing and type of the first composite event: acute heart failure hospitalization, symptom-driven aortic valve replacement, or death. In panel **D**, the black dashed line indicates censoring without an observed composite event. The four patients were selected randomly to illustrate the range of observed FS patterns and follow-up statuses, including each component of the composite endpoint and censoring.

**Figure 3:**
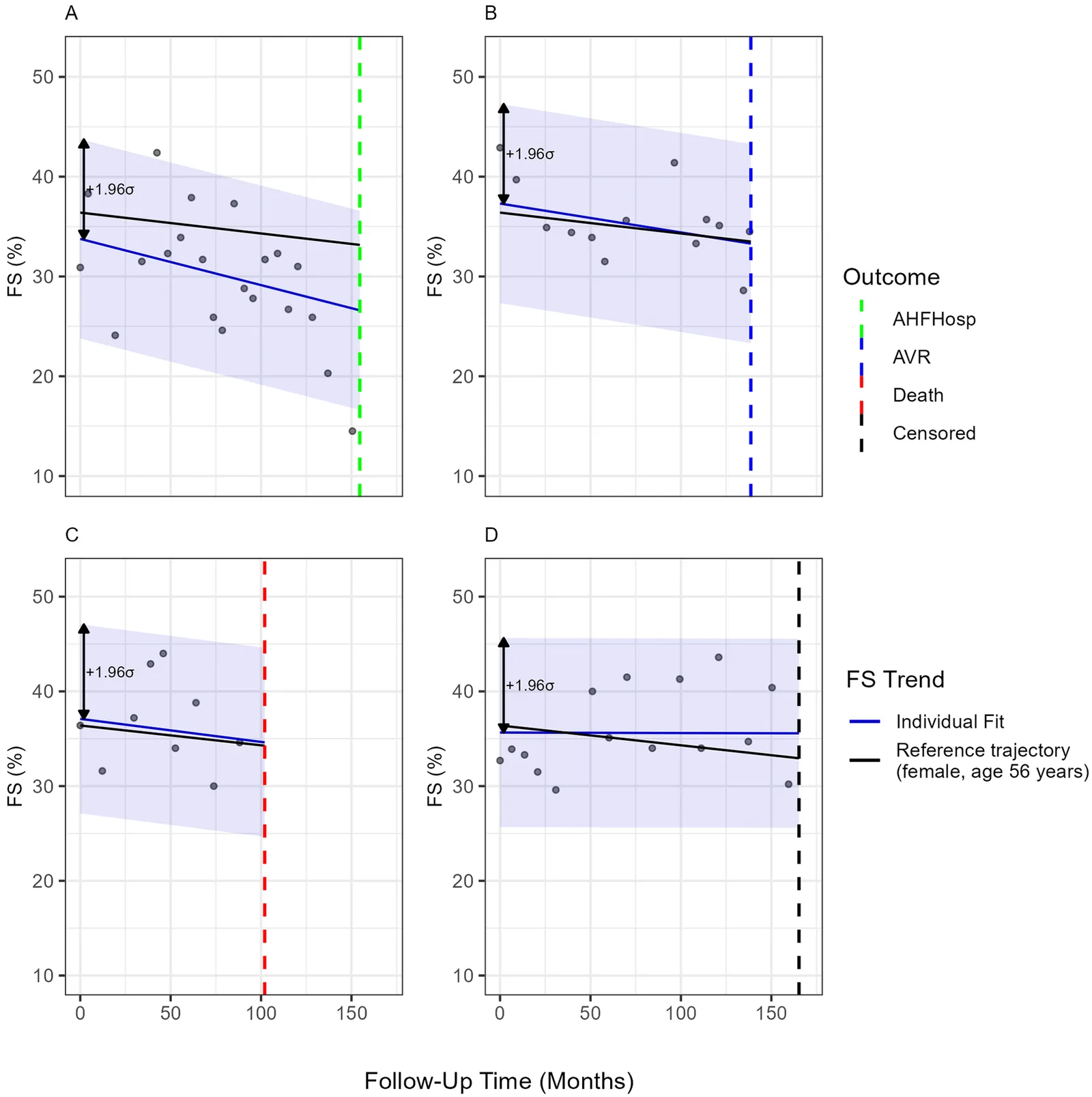
Model-estimated fractional shortening trajectories for the four illustrative patients. Observed FS measurements are shown as points. The blue line is the subject-specific FS trajectory estimated by the longitudinal submodel of the joint model, and the black line is the fixed-effect reference trajectory for a female patient aged 56 years (the cohort median age at onset). The shaded band shows residual measurement variability around the fitted subject-specific trajectory, calculated as the fitted trajectory ±1.96 residual standard deviations. The vertical dashed lines indicate the timing and type of the first composite event in panels **A–C** and censoring without an observed composite event in panel **D**, as defined in Figure 2. These are the same illustrative patients shown in Figure 2.

The survival submodel in Table 3.II estimates how patient age at onset, sex, and current FS level (predicted from the longitudinal submodel) are associated with the hazard of experiencing the composite event. The coefficients presented correspond to effects on the log-hazard scale. Exponentiation of these coefficients yields hazard ratio (HR) estimates. The results show that lower FS values are significantly associated with increased event risk. Specifically, the coefficient of −0.12 for FS implies a HR of approximately 1.13, indicating a 13% higher risk of experiencing the composite outcome per 1% decrease in FS. A 4% decrease in FS corresponded to an HR of 1.62. Age at onset also showed a statistically significant effect, with HRs of 1.05 per year (5% higher risk), 1.28 per 5 years, and 1.65 per 10 years. Sex was not significantly associated with the risk of experiencing the composite event.

### 3.3 Joint model using LVESD as the longitudinal variable

Table 4 summarizes the joint model results with LVESD as the main echocardiographic measure. For female patients aged 56 years, the predicted LVESD at baseline TTE was 34.67 mm. For male patients of the same age, the corresponding predicted LVESD was 37.89 mm. Over time, LVESD showed a small but statistically significant increase of 0.01 mm/month, suggesting a gradual widening of the left ventricle during follow-up. Age at onset was not meaningfully associated with LVESD levels. However, male patients had significantly higher LVESD values than females, by an average of 3.2 mm. The typical variability in individual LVESD measurements around the expected trend, represented by the sigma value, was estimated at 3.38 mm.

**Table 4:** Joint model for LVESD.

Variable	Mean	StDev	2.5%	97.5%	*P*
	I. Longitudinal submodel results				
Female patients aged 56 years at onset (mm)	34.67	1.28	32.08	37.10	<0.01
Follow-up time (months)	0.01	0.00	0.00	0.02	0.01
Age at onset, centered at 56 years	−0.01	0.03	−0.07	0.04	0.66
Male sex (vs. female)	3.22	1.41	0.45	6.01	0.02
Residual SD (sigma)	3.38	0.07	3.25	3.51	<0.01
	II. Survival submodel results				
Age at onset (Years)	0.05	0.01	0.02	0.08	<0.01
Male sex (vs. female)	−0.58	0.42	−1.34	0.29	0.18
Current LVESD (mm)	0.06	0.02	0.02	0.10	<0.01

The survival submodel in Table 4.II shows that patients with larger LVESD values were at higher risk of experiencing the composite outcome, with a HR of 1.06 per 1 mm increase in LVESD (6% higher risk) and 1.20 per 3 mm increase.

Age at onset was also a significant factor, with an identical effect to that in the FS-based parallel analysis. As with the FS analysis, there was no meaningful difference in risk between male and female patients.

### 3.4 Joint model using LVEDD as the longitudinal variable

Table 5 summarizes the LVEDD joint model. For female patients aged 56 years, the predicted LVEDD at baseline TTE was 53.85 mm. For male patients of the same age, the corresponding predicted LVEDD was 58.04 mm. The results indicate that, on average, LVEDD remained stable over time, as the effect of follow-up duration was close to zero and not statistically significant. This non-significant time effect provides no evidence of a consistent population-level linear change in LVEDD during follow-up. LVEDD was not meaningfully associated with age at onset. Male patients had significantly higher LVEDD values than females, by about 4.2 mm on average. The typical variability in individual LVEDD measurements around the expected trajectory of each patient, represented by the sigma value, was estimated at 3.38 mm.

**Table 5:** Joint model for LVEDD.

Variable	Mean	StDev	2.5%	97.5%	*P*
	I. Longitudinal submodel results				
Female patients aged 56 years at onset (mm)	53.85	1.24	51.40	56.24	<0.01
Follow-up time (months)	−0.01	0.00	−0.01	0.00	0.14
Age at onset, centered at 56 years	−0.04	0.03	−0.09	0.02	0.18
Male sex (vs. female)	4.19	1.36	1.50	6.80	<0.01
Residual SD (sigma)	3.38	0.07	3.25	3.51	<0.01
	II. Survival submodel results				
Age at onset (Years)	0.05	0.01	0.03	0.08	<0.01
Male sex (vs. female)	−0.58	0.43	−1.35	0.33	0.19
Current LVEDD (mm)	0.06	0.02	0.01	0.10	0.02

The findings of the survival submodel in Table 5.II closely mirror those from the LVESD model: larger LVEDD values were associated with a higher risk of experiencing the composite outcome, with an HR of 1.06 per 1 mm increase in LVEDD (6% higher risk) and 1.20 per 3 mm increase (20% higher risk). Age was again a significant predictor, with an identical effect to that observed in both LVESD- and FS-based models. Consistent with those models, no meaningful difference in risk was observed between male and female patients.

## 4 Discussion

In our study, we revisited the value of scrutinizing repeated simple 2D echocardiographic measurements, as advocated by the American and European guidelines. We evaluated this in a modern real-world scenario of long-term clinical follow-up of patients with significant AR, using a large retrospective database from a tertiary medical center. Substantial noise was introduced by technical variability, as TTE studies were performed by different cardiac sonographers on different commercial echo machines. In addition, patients underwent varying numbers and frequencies of TTE studies over the follow-up period, further contributing to heterogeneity. Nevertheless, this variability also allowed us to test the robustness of widely used real-world practice tools in a representative clinical setting. By fitting separate joint models for FS, LVESD and LVEDD, we evaluated the measure-specific association with outcome. The longitudinal results of all the models were consistent with the overall variability observed in the repeated measurements, as reflected by the large variability estimates around each patient's assessed trajectory.

In separate joint models, all three echocardiographic measurements (LVESD, FS, and LVEDD) were significantly associated with an increased risk of the composite adverse outcome. A modest but consistent population-level longitudinal trend was observed for LVESD (increase) and FS (decline), whereas LVEDD showed no consistent trend over time. The observed LVESD increase rate was similar to that reported by Bonow et al. in AR patients who developed death, symptoms, or left ventricular dysfunction (8).

Based on the models intercepts, male patients had, on average, larger hearts than female patients (about 3 mm greater LVESD and about 4 mm greater LVEDD), consistent with known physiological gender differences (16). However, gender was not associated with differences in the composite risk in any of the joint models.

Age at onset was a significant predictor for the composite event in all joint models, with a consistent hazard ratio of 1.05 per additional year, or 1.28 per five additional years. Despite a higher risk of post-operative mortality and complications in elderly patients (17), this finding supports AVR consideration in older patients who meet guideline-based indications (i.e., LVESD increase or LVEF decline) and lack significant comorbidities.

Because FS is calculated from LVEDD and LVESD, it is not an independent structural measure. Rather, as a relative measure of systolic shortening, FS reflects the relation between end-systolic and end-diastolic dimensions and may provide a complementary functional summary to either absolute diameter alone. We modeled FS, LVESD, and LVEDD separately because of their mathematical dependence. Accordingly, the hazard ratios should not be compared directly across measures: they were estimated in separate models and are expressed on different scales. The numerical magnitude of the HR per 1% decrease in FS therefore does not establish a stronger association with outcome than the HRs per 1-mm increase in LVESD or LVEDD. A formal assessment of whether FS adds prognostic information beyond the two diameters would require a dedicated multivariable prediction analysis.

Although TTE basal linear LV diameters are useful for prognosticating patients with chronic AR, they may come at the risk of poorly capturing the true LV remodeling and eccentric LV dilation that commonly occurs (18, 19). The LVESD is a unidimensional estimate of LV end-systolic volume, and LVEDD an estimate of LV end-diastolic volume, with only moderate correlation between the linear dimensions and volumes in AR patients (19–21). The modest but statistically significant increase in LVESD observed over long-term follow-up, in contrast to the absence of a consistent change in LVEDD, may reflect the different physiological determinants of these measures. Whereas LV end-diastolic volume largely reflects preload, LV end-systolic volume is influenced by both preload and systolic performance and may therefore better reflect LV remodeling in AR patients (22, 23). In fact, several studies showed that LV end-systolic volume (or indexed volume) predicts postoperative outcomes, survival, and symptom progression better than LV end-diastolic volume in AR (19, 20, 24). It has been shown that linear dimensions measured at the midventricular level, rather than the traditional basal level, are more closely correlated with volumetric measurements of LV size, and LVEF (18), but midventricular dimensions were not recorded systematically in our study.

Based on our results, we evaluated the clinical relevance of the current guideline recommendation to perform AVR when LVEDD exceeds 65 mm (2, 3). Using the fitted longitudinal model, we predicted individual LVEDD trajectories over a 10-year follow-up period, which reflects a reasonable extension of the observed median follow-up of 7.75 years. Model-based predictions indicated that only 8 out of 140 patients (5.7%) were expected to exceed the 65 mm threshold at any point during this period. Of these, four patients had persistently elevated LVEDD values above 65 mm, while three patients started with values above 65 mm but showed decreasing trajectories over time. Importantly, only one patient crossed the 65 mm threshold during follow-up, representing upward progression. These findings question the applicability of a fixed LVEDD cutoff as a standalone intervention criterion. Similarly, in a recent study of 365 patients with severe AR who underwent AVR, de Meester et al. found no difference in post-AVR survival between those with LVEDD below vs. above 65 mm (7). However, their design and outcome measure (post-operative mortality) differed from ours (composite endpoint pre-AVR), limiting direct comparability.

FS became a popular measure of LV function with the advent of M-mode echocardiography in the late 70's (25, 26). In a small old study of patients with AR, FS was found to decrease to a lesser extent in asymptomatic patients compared with those who developed dyspnea (27). FS has well known limitations, as it is a linear parameter which may not accurately represent overall cardiac function in patients with regional wall motion abnormalities or abnormal ventricular activation (16). However, it correlates with LVEF and, in this study, served as a surrogate for LVEF in AR patients since precise quantitative LVEF was unavailable in most TTE studies in our database.

The modest decline in FS observed over time, together with the increased adverse risk associated with FS decrease resonate with the known LVEF decline and its prognostic value in AR patients in literature (4, 8, 28, 29). These findings also align with a 1980 study showing that preoperative FS < 25% identified patients at risk of post-AVR death related to congestive heart failure (9). Interestingly, although 12 patients (8.6%) in our study had reduced LVEF, the median FS on baseline TTE was 35%. This value is within normal range according to old data for healthy subjects of similar age (25, 26), and is higher than the average FS of 30% reported in a prior study of 50 patients with AR (9).

### 4.1 Limitations

A major limitation of our study is its long-term retrospective nature, which is inherently associated with variability in the characteristics of TTEs performed related to clinical and sonographer factors. The joint modeling approach we utilized in this setting enabled us to meet the aforementioned challenges.

Although the dataset contained many potential variables, the relatively small sample size (140 patients) limited the complexity of the statistical models. Including more variables would have made them prone to overfitting and unstable results. Therefore, the models included only a few covariates and did not jointly model multiple time-dependent variables.

Important parameters for AR assessment and management, including MRI-derived volumes and LVEF, as well as echo-derived biplane method-of-disks volumes and LVEF and midventricular diameters (which, as mentioned earlier, correlate better with volumetric measurements of LV size), were unavailable for many patients in our cohort. In most patients in our database, only qualitative (categorical) LVEF was recorded. Therefore, we used FS as a continuous, though less precise, surrogate measure of LV systolic function instead of quantitative LVEF.

## 5 Conclusion

In this real-world cohort of patients with moderate-to-severe or severe AR, LVESD increased modestly but significantly during long-term follow-up, whereas LVEDD showed no consistent population-level change. Nevertheless, higher LVESD and LVEDD, as well as an older age, were associated with increased risk of the composite outcome. FS, used as a surrogate for LVEF, declined modestly but significantly over time, and lower FS values were also associated with higher risk. Although men had larger average LVESD and LVEDD values than women, sex was not associated with adverse composite outcomes.

Taken together, our findings show that, in separate joint models, LVEDD, LVESD, and FS were each associated with adverse outcomes, supporting their prognostic role in the longitudinal assessment of patients with AR. However, the relatively small effect sizes and considerable variability in individual trajectories indicate that AVR decisions should be based on a comprehensive clinical assessment rather than changes in these parameters alone. The model predicted that only a small proportion of patients would surpass the 65 mm LVEDD threshold during follow-up, and in most of these cases this did not reflect a progressive increase over time. These findings suggest that this criterion may have limited utility as a sole trigger for intervention in routine follow-up.

## Data Availability

The raw data supporting the conclusions of this article will be made available by the authors, without undue reservation.
